# The Future of Health Care: Protocol for Measuring the Potential of Task Automation Grounded in the National Health Service Primary Care System

**DOI:** 10.2196/11232

**Published:** 2019-04-09

**Authors:** Matthew Willis, Paul Duckworth, Angela Coulter, Eric T Meyer, Michael Osborne

**Affiliations:** 1 Oxford Internet Institute University of Oxford Oxford United Kingdom; 2 Machine Learning Research Group Department of Engineering Science University of Oxford Oxford United Kingdom; 3 Health Services Research Unit Nuffield Department of Population Health University of Oxford Oxford United Kingdom; 4 School of Information The University of Texas at Austin Austin, TX United States

**Keywords:** qualitative research, supervised machine learning, automation, interdisciplinary research, task performance and analysis

## Abstract

**Background:**

Recent advances in technology have reopened an old debate on which sectors will be most affected by automation. This debate is ill served by the current lack of detailed data on the exact capabilities of new machines and how they are influencing work. Although recent debates about the future of jobs have focused on whether they are at risk of automation, our research focuses on a more fine-grained and transparent method to model task automation and specifically focus on the domain of primary health care.

**Objective:**

This protocol describes a new wave of intelligent automation, focusing on the specific pressures faced by primary care within the National Health Service (NHS) in England. These pressures include staff shortages, increased service demand, and reduced budgets. A critical part of the problem we propose to address is a formal framework for measuring automation, which is lacking in the literature. The health care domain offers a further challenge in measuring automation because of a general lack of detailed, health care–specific occupation and task observational data to provide good insights on this misunderstood topic.

**Methods:**

This project utilizes a multimethod research design comprising two phases: a qualitative observational phase and a quantitative data analysis phase; each phase addresses one of the two project aims. Our first aim is to address the lack of task data by collecting high-quality, detailed task-specific data from UK primary health care practices. This phase employs ethnography, observation, interviews, document collection, and focus groups. The second aim is to propose a formal machine learning approach for probabilistic inference of task- and occupation-level automation to gain valuable insights. Sensitivity analysis is then used to present the occupational attributes that increase/decrease automatability most, which is vital for establishing effective training and staffing policy.

**Results:**

Our detailed fieldwork includes observing and documenting 16 unique occupations and performing over 130 tasks across six primary care centers. Preliminary results on the current state of automation and the potential for further automation in primary care are discussed. Our initial findings are that tasks are often shared amongst staff and can include convoluted workflows that often vary between practices. The single most used technology in primary health care is the desktop computer. In addition, we have conducted a large-scale survey of over 156 machine learning and robotics experts to assess what tasks are susceptible to automation, given the state-of-the-art technology available today. Further results and detailed analysis will be published toward the end of the project in early 2019.

**Conclusions:**

We believe our analysis will identify many tasks currently performed manually within primary care that can be automated using currently available technology. Given the proper implementation of such automating technologies, we expect considerable staff resources to be saved, alleviating some pressures on the NHS primary care staff.

**International Registered Report Identifier (IRRID):**

DERR1-10.2196/11232

## Introduction

Automation technologies are rapidly changing employment practices across many sectors of the UK economy. The progress of advancements during the digital age has seen new technologies replacing and augmenting human labor in a diverse range of tasks, reshaping the experience of millions of customers and employees. In this protocol, “Automation” is defined as applications of robotics, artificial intelligence, machine learning, machine vision, and similar emerging and mature digital technologies that will allow human work to be substituted by computer capital. It is within this scope that our work aims to understand the state-of-the-art automation in the primary health care sector. A recent example of applying automation technologies to augment or replace human labor is Amazon, a company that recently launched a grocery store—Amazon Go—that uses computer vision to eliminate the role of the cashier, which relates to over 3.5 million people in the United States [1,2]. In addition, Amazon and others also harness intelligent material-moving robots to work alongside the 2.5 million freight and stock hand laborers in warehouses and commercial buildings [3]. Looking ahead, continued progress in state-of-the-art automation technologies will cause further disruption to workers in knowledge- and information-based occupations, who were previously thought to be less susceptible to automation. However, despite widespread concern regarding new technology replacing jobs or how technology will change the structure of jobs, we lack detailed real-world evidence about what can and cannot be automated at the task level; tasks, not entire occupations, are automated. This lack of understanding can be confusing and dangerous for policymakers who want to set effective policies to mitigate the consequences and foster potential benefits.

Dominant frameworks for measuring automation have previously focused on different “types” of occupations and the skills that are required to perform them [4-6]. These studies conclude that occupations with so-called “routine” tasks are the most susceptible to automation, and specifically, manual occupations are easier to automate than cognitive or knowledge-based occupations. Researchers at the University of Oxford analyzed the US Department of Labor Occupational database (O*NET) and found that 47% of US employment is highly susceptible to automation over the next few decades [4]. They propose a probabilistic machine learning approach using numerical occupation features that represent “bottlenecks” to automation and analyzed over 700 occupations, producing an estimate of the *probability of automation* for each. Multiple follow-up studies applied these probabilities to other countries’ employment data, assuming an occupation’s risk of automation is comparable across countries. Deloitte reported [7] that 35% of current UK employment is at high risk of becoming automated over the same time period. Furthermore, a paper from the Bruegel Think Tank [8] estimated the share of jobs at high risk across Europe to range between 45% to >60%, with southern European workforces (eg, Portugal and Romania) facing the highest probability of potential automation.

From these studies, health care–oriented roles are often estimated to be at low risk of automation. This is, in part, because many health care tasks require a high level of skills that align with the bottlenecks to automation identified [4], such as assisting and caring for others, manual dexterity, social perception, originality, negotiation, and persuasion. A secondary reason for these low-risk estimates is a general lack of empirical data describing work practices, work flows, and the skills required to perform many health care roles, in what is a largely interrupt-driven environment containing many exceptions and social negotiation.

Automated technologies are often speculated to target or displace vulnerable, low-skilled workers. Health care is one of the few economic sectors where automation is seen as an opportunity to address pressures [9,10]. Specifically, the UK National Health Service (NHS) primary care system in England currently faces numerous building pressures such as staff shortages, increased workloads, increased demand, reduced budget, skill shortages, and decreased patient consultation time [10-12]. Generally, automation may address some of these pressures. However, there is a potential threat that through increased automation of tasks, the roles performed by health care staff will need to be reconfigured as described previously [13], which may ultimately affect patients’ relationship with their general practitioner and the level of care provided.

A key aspect of our approach is to start with tasks, rather than occupations, to understand what *technically* can be automated and how an occupation’s work might be impacted as a result. By collecting granular task-level data, we capture a more accurate effect of automation, since it is tasks, rather than entire occupations, that are automated by new technologies. This approach also provides the most valuable real-world policy insights, with recommendations over entire workflows, potentially saving considerable resources.

The future health sector will undoubtedly involve automation of routine tasks such as scheduling or laboratory test-review tasks; it is also likely to involve technologies that are uniquely developed and still in their infancy. The current applications of automation in health care are a rich and well covered topic with decades of history. The literature includes examples such as electronic medical records, personal health records, remote test ordering and repeat prescriptions, check-in and booking systems, patient access to appointment systems, telehealth and telemedicine systems, physician order entry, clinical decision–support systems, much of the pharmacist’s work [14], automation of data collection from patients in the waiting room [15], and a reduction in provider-to-provider communication [16]. Additionally, numerous different software systems have an element of automation; for example, computerized physician order entry (CPOE) is a decade-old technology that has helped automate workflows such as requesting lab work, checking for allergies, and electronic prescribing [17]. A sophisticated CPOE system can automate some, if not most, of a clinician’s work with increasing efficiency. However, it is not completely automated, and a human clinician is still required to keep notes, converse with colleagues, work directly with the patient, reference materials, and likely work in other systems besides CPOE. This example of CPOE systems and similar support systems exemplifies the core of our study: There are automated and partially automated systems to be found throughout primary care. Nonetheless, there is a lack of understanding of the effects of automation to provide further policy and workflow recommendations. Furthermore, the implementation of automation technologies on the assumption that they will *always* provide typical benefits associated with technology may be a false promise, as many information system–implementation projects in health care run over budget, fail to deliver expected results, and demand compromises; these projects also require significant amounts of organizational support and maintenance [18].

Clinical decision–support systems (CDSSs) are similar information systems to computerize physician order-entry systems. A CDSS is an information system designed to, as the name suggests, support clinicians through the process of making decisions on a diagnosis or recommending a treatment or change in therapy. The type of system can range in complexity, depending on what is automated from the clinician’s decision-making tasks. A simple CDSS will only check the input from the clinician to confirm that the input is valid and within the range of the specified field, producing an error or notification if the data are invalid. Automated CDSSs are developed for specific clinical specializations and involve the use of data models, standardized medical knowledge ontologies, and other medical and clinical knowledge along with data inputs from electronic medical records to support diagnoses based on the system’s guidelines. Complex CDSSs use a series of computational, data mining, and statistical methods to support complex reasoning on classifications of disease, predictions of a disease, or patient concern [19]. Although CDSSs benefit the clinical decision-making process and most studies show that these systems provide benefit, they are easily susceptible to automation bias—the phenomenon where clinicians overly rely on decision systems to the detriment of their own reasoning [20]. Thus, it is important to be mindful of the potential detriments of automation. Understanding the full range of tasks that can be automated and how automation of certain tasks influences the overall work of the clinician is an important step to preventing automation bias.

Prior work also exists in the use of mobile robotics in health care. Areas such as robot-assisted logistics, telepresence and companion robots, education and communication robots, motivational persuasive robots, ageing society robots, and home-assistance robots, all introduce automation health care [21].

Robotic systems are in their infancy, and although many novel approaches are under development for the health care domain, our study does not focus on recommending what specific *type* of technology will or can automate a task. Instead, we seek to understand to what extent health care tasks *technically* can be automated using currently available technology and to interpret and disseminate the effects of this automation in the health care domain.

We have organized the protocol for this study along two aims: (1) to observe and collate a comprehensive understanding of what occupations and tasks occur in primary health care practices and (2) to use expert knowledge of the current state-of-the-art automation technologies as a guide to estimate what tasks and work practices are automatable.

## Methods

### Design

Our approach constitutes a multimethod research design [22]. Aim 1 uses multiple techniques for gathering qualitative data based on the observed tasks performed by all occupations in primary care. Aim 2 employs a survey and quantitative machine learning framework to analyze the empirical structured qualitative data gathered for aim 1.

A critical part of the problem we intend to address is a formal framework for measuring task-level automation, which is lacking in the literature. This is compounded in the health care domain by a general lack of detailed, health care–specific occupation and task data for good policy insights. Therefore, our first aim focuses on addressing this lack of data by collecting qualitative, high-quality, detailed task-specific data from multiple UK primary health care practices. The second aim then proposes a formal, quantitative framework for probabilistic inference of *task-* and *occupation*-level automatability.

**Figure 1:**
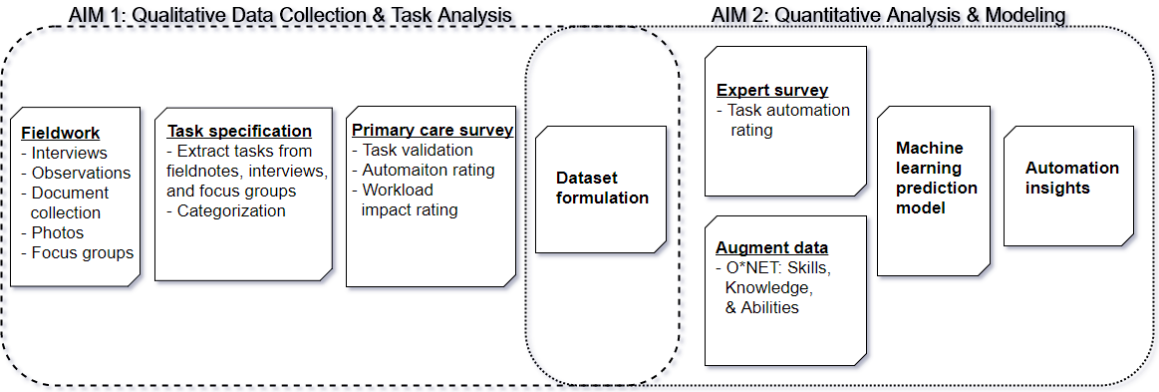
Study design. O*NET: US Department of Labor Occupational database.

Figure 1 presents a graphical view of the two aims, and specifically, how they interact in the dataset-formulation stage. The project can be summarized by the following key stages:

- Fieldwork: Detailed qualitative observational work over a period of 12 months while visiting primary care practices in England.
- Task specification: Qualitative analysis to categorize observed work into a formal concept of a *task* within primary care. As a basic unit of work, a task contains a detailed description of work performed and many indicator variables.
- Primary care survey: Conduct an online survey of primary care staff, aimed at understanding and validating task specification and the tasks that most impact daily workloads.
- Dataset formulation: A process of recording each observed task performed by each occupation in a practice and formulating a matrix of observed occupation-task pairs.
- Expert survey: Conduct a large online survey on automation, including top academics and industry experts in machine learning, robotics, and artificial intelligence, to rate how automatable specific tasks are today (not restricted to the health care domain).
- Augmenting the dataset: Our primary care occupation-task dataset, meticulously derived in aim 1, is augmented with numeric attributes available from a publicly available occupational survey produced for the US Department of Labor called the O*NET database.
- Machine learning predictions: The numeric O*NET attributes describing the skills, knowledge, and abilities each task requires are used as input in a machine learning model, trained on the tasks where expert estimates are available (from the survey), in order to predict the automatability of health care tasks.
- Insights: Analysis performed on the automatability of tasks in primary care, the occupations that involve them, aligned with detailed qualitative analysis to form policy recommendations to aid current working practices.

Each of these stages is further detailed in the following sections for aim 1 and aim 2.

### Aim 1

#### Process

We first address the general lack of detailed data on the topic of automation in the NHS health system. Our aim is to observe work practices and collect data for a comprehensive list of tasks performed by each occupation in NHS primary care. This collection of detailed and rich data is guided by interviews, document collection, photographs, detailed field notes, and occupation shadowing. These observational data are qualitatively analyzed and organized into a formal dataset that supports the second aim of the project: Analyzing the data using machine learning techniques to infer automatability. The dataset created in aim 1 is validated using focus groups, where tasks performed by an occupation are presented and discussed in person.

The qualitative work detailed in aim 1 of this protocol complies with the requirements of the Department of Health Research Governance Framework for Health and Social Care 2005 in the United Kingdom and is approved by both a research ethics committee and the NHS Health Research Authority. No patient data are collected as part of this research.

#### Fieldwork

To understand the work practices of all primary care staff, from partner general practitioners to receptionists, we employ an ethnographic method to observe situated practices, ask questions, gather documents, write detailed field notes, and catalogue each occupation with as much clarity as possible. Time spent on site at each health center ranges from 3 days to over a week. Prior to starting fieldwork, the field researcher works with practice staff to build a schedule, where time can be made available with each occupational type. Since this project is interested in the *tasks* each *occupation* performs, we do not need to observe every member of the staff, but only a representative subset if there are multiple employees of each occupation type. When developing this schedule, we will also make time for the field researcher to attend general practitioner meetings, chronic disease clinics, and other special events that showcase other occupational tasks of primary care. To date, six practices have been recruited, with task data collected on every occupation type in each practice.

The field research focuses on four streams of data collection:

1. Observation of day-to-day work and tasks performed by staff members. This includes asking detailed questions and behavioral queries to understand specific skills required to accomplish tasks, the description of specific computer use and software configuration, or the specific order in which filing must be performed (ie, any details about identified routine tasks).
2. Collection of documents such as training manuals, job-description documents, policy and protocol manuals, and other organizational documents that describe work tasks and how the practice is to be run. This can extend to photographs of documents or information scattered throughout the practice to understand how work and tasks are documented and distributed.
3. Photographs of work spaces and manual physical tasks in the general practice.
4. Audio-recorded discussions of work processes or tasks taking place and any specific required skills necessary to perform day-to-day tasks.

#### Focus Groups

At the end of the field researcher’s observations and initial data collection, a focus group is conducted with all primary care staff at the facility, given staff availability. The focus group, while providing additional data, serves as a validation technique for the collected data. During the focus group, the field researcher presents their representation and descriptions of tasks to each occupation in turn to achieve an accurate representation. Additional information can be added to the collected data at this stage through conversations with the individual workers to best portray each occupation’s work.

In addition to task validation, the focus group allows for discussion of health care professionals’ perceived benefits, opportunities, and challenges to automation of work in primary care. The field researcher also presents several different scenarios that involve the automation of different types of work in the health center. These scenarios are intended to generate discussion between participants in the focus group about potential changes in work due to automation.

#### Task Validation and Workload Measurement Survey

The second validation technique utilized in this study is the distribution of a survey to support the accuracy of task descriptions for each occupation and to provide a rating of how automating a task would impact individual’s workload. The survey aims to augment the tasks gathered throughout the fieldwork, focus groups, and interviews described above. Survey respondents are shown the set of tasks that are performed in their occupation. For a subset of tasks, the survey asks the question, “If it were possible to fully automate the above task, i.e. entirely performed by a computer or a collection of technologies, how would it influence your daily workload?” The response options include “I do not perform this task,” “There would be no change in my workload,” “There would be little change in my day to day workload, and would not save much time,” “Automating this task would provide me time to work on other tasks in my workload,” and “Automating this task would eliminate a core aspect of the work identified in my job description.” The survey data collected are used to augment the detailed observation task dataset created throughout the aim.

### Aim 2

#### Process

In the second part of the project, we develop a formal, quantitative method for inferring the automatability of all health care tasks observed during aim 1. First, we augment the collected health care occupation-task dataset with existing high-dimensional data about skills, knowledge, and abilities required to perform each task (120 numeric attributes). Second, a large and comprehensive survey of machine learning, robotics, and artificial intelligence experts is conducted to elicit expert estimates regarding the current state of automation of real-world tasks, not specifically restricted to the health care domain. Third, these estimates are used to train a probabilistic machine learning model to identify patterns connecting task automatability to the occupation and task characteristic attributes. The three steps—augmentation of task dataset, automation of expert survey, and development of the machine learning model—are discussed in detail below.

#### Augmenting Task Dataset

**Figure 2:**
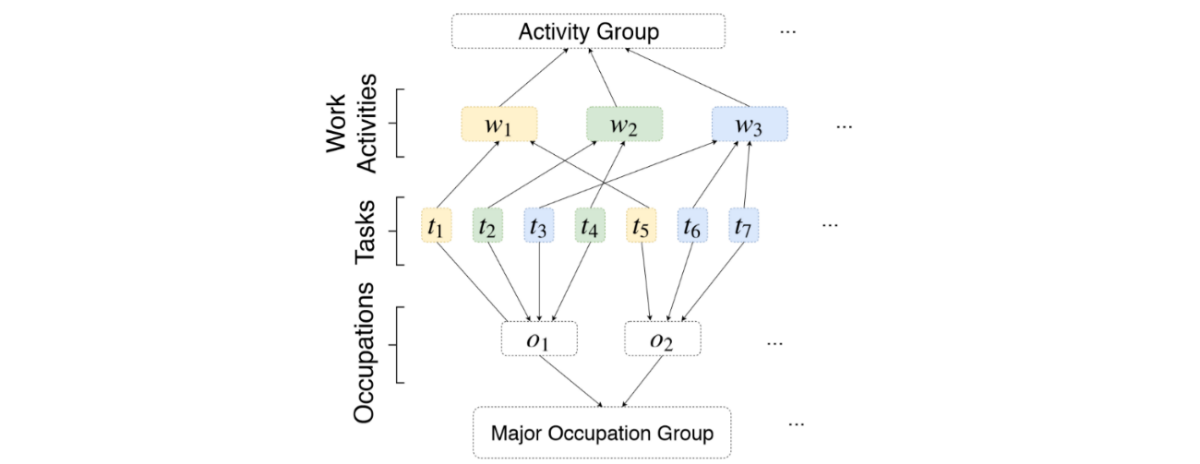
Simplified overview of O*NET database architecture, representing occupations (o_1_, o_2_), tasks (t_1_...t_7_), work activities (w_1_, w_2_, w_3_), and major occupation groups and activity groupings. O*NET: US Department of Labor Occupational database.

The detailed observational and qualitative health care–specific data captured in aim 1 is transformed into a matrix of occupational roles and tasks performed, with each row representing a unique occupation-task pair plus indicator variables. We then augment these identified occupation-task pairs with numeric attributes from a publicly available occupational survey produced for the US Department of Labor: O*NET 2016 database. O*NET provides key features of an occupation as a standardized and measurable set of variables as well as open-ended descriptions of specific tasks each occupation performs; its strengths and weaknesses are reviewed in detail in a previous study [23]. The database contains information on more than 1000 US occupations using a modified form of the Standard Occupation Classification system, comprising over 2000 detailed work activities and nearly 20,000 individual occupation-specific tasks arranged in a hierarchical structure. A simplified hierarchy of the O*NET taxonomy is presented in Figure 2.

**Figure 3:**
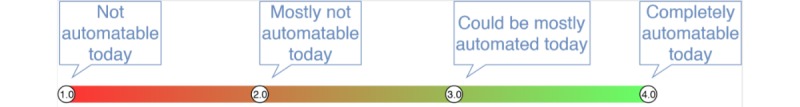
Our continuous scale of automatability.

The O*NET occupational variables include 35 skill attributes such as “coordination,” “critical thinking,” and “time management”; 33 knowledge attributes such as “mathematics,” “clerical,” and “sales and marketing”; and 52 abilities such as “depth perception” and “speech recognition.”

The variables described as “bottlenecks to automation” in previous literature [4] are a subset of the 120 O*NET variables used in our study. Further, in a recent report by McKinsey Global, 18 of the O*NET variables were used to represent work activities [23]. We will not manually select a subset of the variables in this work. The numerical attributes are designed to provide an accurate representation of an exemplar employee within each O*NET occupation, where each occupation is *also* represented by the collection of tasks they are required to perform. We assume that an occupation’s skills, knowledge, and abilities inform those needed to perform the occupation’s list of tasks. Next, we aggregate the occupation variables into work-activity variables by taking a weighted average of an activity’s tasks, normalizing it over the combined weight of the task’s relative importance to its occupation and work activity.

We manually match the observed health care tasks to their corresponding “work activities” within the O*NET hierarchy, allowing for a one-to-many weighted mapping. This allows the observed health care tasks to be augmented with high-quality O*NET variables related to the level of skills, knowledge, and abilities required to perform such tasks. The result of these manipulations is that each observed health care occupation task is represented by a vector of 120 numeric attributes, which are vertically stacked to become a training data matrix for our proposed machine learning model.

#### Automation Expert Survey

The second step is elicitation of expert knowledge of state-of-the-art automation technologies. In order to obtain estimates on how automatable our health care–specific tasks are, we survey machine learning, robotic, and artificial intelligence experts at the forefront of research and commercially available technology. The survey is designed such that each participant is presented with five O*NET occupations (chosen to be representative of the feature space, with an emphasis on high employment, and hence, familiar occupations). Survey participants rate how automatable the five “most important” tasks are (task importance is relative to occupation, as defined in O*NET). The survey asks the question, “Do you believe that technology exists today that could automate these tasks?” Participants rate each task with one of the following options: 0, Unsure; 1, Not automatable today; 2, Mostly not automatable today (human does most of it); 3, Could be mostly automated today (human still needed); and 4, Completely automatable today.

Our demographic is specifically technology experts, as opposed to health care experts, because we believe that annotating basic tasks requires little-to-no subject matter knowledge. If respondents feel any doubt in their ability to assess the automatability of a task, they can select the “Unsure” option.

We combine each task’s multiple expert labels using Independent Bayesian Classifier Combination (IBCC), a principled Bayesian approach to combine multiple classifications [24]. IBCC creates a posterior probability over labels that reflect individual labeler’s tendencies to agree with other labelers over the ultimately chosen label values. We then average the IBCC task scores into their task’s work activities (corresponding to the tasks’ parent in the O*NET hierarchy; Figure 2). A score of 4 represents a fully automatable work activity, and a score of 1 represents an activity that cannot be automated using currently available technology (Figure 3).

We believe a survey of experts, combined using IBCC, provides a robust ground truth estimate to what extent activities are automatable using currently available technology. One important note is that the survey results provide a measure of what *can* be automated using technology, with no prediction of future technological advancements (ie, not what necessarily *will* be automated, given technology uptake or societal pressures).

#### Development of the Machine Learning Model

Finally, we plan to use a machine learning framework to learn functional mapping between the skills/knowledge/ability feature vectors (the 120 O*NET attributes) of a work activity and the ground truth automation scores elicited from our expert survey combined using IBCC. Gaussian processes [25] are a modeling tool that have a natural advantage in this scenario and offer advantages to policymakers, such as providing formal estimates of uncertainty within the model. The algorithm uses the trends and patterns it has learned from labeled data to provide a smoothly varying, probabilistic assessment of automatability as a function of the input variables. For the Gaussian process, this function is nonlinear, meaning that it ﬂexibly adapts to the patterns inherent in the training data. Gaussian processes have been successfully applied to occupation-based data [4,26], personalized electronic health monitoring [27], and patient-risk monitoring [28].

We will train the Gaussian process model on 314 work activities present in O*NET, for which expert labels are available from the survey. We will specifically use the ordinal likelihood function [29] to reflect the nature of having discrete labels but with an ordinal interpretation (“not at all” to “completely” automatable). In brief, we will optimize the Radial Basis Function kernel hyperparameters by minimizing the negative marginal log likelihood [21]. Once trained, the model will allow us to estimate the automatability of “unlabeled” work activities (ie, activities where expert labels were prohibitively difficult to obtain). This is performed using open-source software GPFLow [30]. The Gaussian process model is evaluated based on its ability to predict the automatability of work activities “held out” from the training process. We compare models based on their “tolerance accuracy” score, which is the percent of predictions within 0.5 of the ground truth IBCC survey score. This is a sensible score for our task and allows more flexibility in our multiclass ordinal setting than strict accuracy or average error. We find that the ordinal Gaussian process performs better than other methods such as random fields, neural networks (with ordinal loss), or ordinal regression.

## Results

We have recruited six general practice medical centers as of October 2018. We have started looking at each occupation and their work practices in primary care. We anticipate that the results will be available in early 2019. The initial findings will be disseminated in a report for the project funder, The Health Foundation. Some preliminary findings from the project are presented below.

We have identified 16 unique occupational roles in primary care. These 16 occupations conduct all the work that occurs in primary care and have currently catalogued over 130 unique tasks. In general, each occupation performs 10-20 tasks regularly. For practice staff (nonclinical occupations), there are three to eight tasks that require collaboration of another person to complete. For example, signing off prescriptions or letters, reviewing documents, gathering signatures from multiple people, or entering a portion of data into an electronic system.

Apart from face-to-face meetings and phone calls, we observed that nearly every other task relies on a desktop computer in some way. This heavy use of desktop computers is an important indicator for future automation, since it is likely that software-based automation will be a large driver of further automation such as robotic process automation. For example, most staff in primary care spend the majority of their time interacting with the electronic medical record; however, we have observed that different occupations use the software in different ways.

Going forward, we expect to determine how a task could be restructured, what technologies the task might require, how much time an occupation spends in performing tasks, and how multiple staff collaborate on tasks, all of which highlight important factors used to address the second aim of our research.

Inductive qualitative content analysis has been performed as part of aim 1. The results of this analysis have produced categories that we will continue to build on and plan to use in future analysis. These categories will help us identify work that can be automated or presents a technological challenge to automation. Specifically, we are interested in potential correlations between the identified categories of primary care work and their association with the probability of task automation. This correlation will help us identify categories of work or entire workflows that are closely correlated with high or low probabilities of task automation in order to propose future automated workflow design within the health care domain.

## Discussion

In this protocol, we address two issues: one is an inherent lack of detailed data on task-level work practices of occupations in primary care and the second is the development of a formal representation for estimating task automatability and its impact on health care occupations.

Through this work, we advance earlier research from the University of Oxford [4] on automation and its effects on employment. Specifically, we address the understanding that occupations are unlikely to be automated in their entirety, but their composite tasks may be automated, by taking a task-level approach to modeling automation [13,31,32] and identifying where efficient workflows could arise. We believe this work will inform policy decisions and best practices in primary care on the design and configuration of occupational workloads and tasks in primary care. Specifically, we hope to provide some guidance about where in the general practice surgery work can be automated, what kind of work is most amiable to automation, and what type of skills that cannot be easily automated are important for the health care sector to invest in their future workforce.

There is a common belief that typical health care–related occupations are associated with a low risk of automation. Through our detailed task-level data collection and expert elicitation, we gain clear insight into which tasks are *technically* automatable. In turn, our machine learning model learns which tasks require a human to manually perform them and specific attributes that drive higher or lower automatability estimates using sensitivity analysis.

From our initial fieldwork, we have found that many forms of automation already exist in health care. We observed that these forms of *automation* increase the productivity of human employees; however, they often do not remove tasks entirely. In fact, some of these forms of automation have created *more* work for staff. Although automation has allowed humans to process tasks more efficiently, more administrative work needs to be processed as well. We anticipate that our analysis will inform the design and reconfiguration of work processes in primary health care and lead to recommendations of new automated processes.

## Abbreviations

O*NET: US Department of Labor Occupational database
NHS: National Health Service
CPOE: computerized physician order entry
CDSS: clinical decision support system
IBCC: independent Bayesian classifier combination
